# Supplementary material for: Taste alteration and its relationship with nutritional status among cancer patients receiving chemotherapy, cross-sectional study
Source: PLoS One. 2024 May 9;19(5):e0302990. doi: 10.1371/journal.pone.0302990 (PMC11081316; doi:10.1371/journal.pone.0302990)
Supplement: Attachment — Submitted filename: Response to Reviewers.docx [file pone.0302990.s001.docx]

Editor-in-Chief PLOS ONE Journal

Dear Editors and reviewers,

Greetings

# We would like to submit the revised files of our manuscript title:

**“Taste alteration and its relationship with nutritional status among cancer patients receiving chemotherapy, cross-sectional study.”**

Ref. No.: PONE-D-23-31394

# We kindly thank you for your time and effort invested during the review of our manuscript. We appreciate the comments and suggestions on the submitted version of our contribution. We did all the corrections suggested by the reviewers attached to this letter in point-to-point form, all the corrections have been approved by all authors. The changes in the manuscript were mentioned in the below table also they were highlighted in yellow in the manuscript for easier tracing.

# We hope that the answer letter and the improved version of the paper will clarify open issues and we are looking forward to additional feedback on our revised proposal.

# Kind regards, The authors team.

**Corresponding author:** Dr. Manal Badrasawi

Email: [m.badrasawi@najah.edu](mailto:m.badrasawi@najah.edu)

P.O. Box: 7

Address: Nutrition and Food technology department, Faculty of Agriculture and Veterinary Medicine, An-Najah National University, Nablus, Palestine

Hand phone: +972595095811

| **1** | **Comment** | **Reply** | **Corrections on the manuscript** |
| --- | --- | --- | --- |
|  | 1. In lines 49-50: The author’s state: “and patients exhibiting increased sensitivity to it (6).” In reference 6, authors declare that sweet thresholds significantly declined in patients. Please correct the statement. | Thank you for your comment, and sorry about the confusion related to my statement.  The authors in the reference (6) stated in their results: “throughout treatment in BC patients, sweet threshold significantly declined”, and stated in their conclusion: “during treatment, sweet taste sensitivity increased while other tastes were unaffected”. Both statements are in line with each other and high sensitivity to a certain taste indicate that the taste threshold is declined since taste threshold is “the lowest concentration of a tastant that can be detected” and taste sensitivity is “the minimum concentration at which the subject is able to perceive a specific taste quality”. | The following paragraph has been edited (highlighted in yellow):  And patients exhibiting increased sensitivity to sweet taste, accompanied by a significant decrease in sweet thresholds [6]. |
|  | 2. The authors evaluate different parameters by using specific scales or tools: TAs were evaluated using the chemotherapy-induced taste alteration scale (CiTAS), the malnutrition universal screening tool (MUST) was used for nutritional screening, the antineoplastic side effects scale (ASES) was used for subjective assessment of chemotherapy side effects, and the Charlson comorbidity index (CCI) was used for comorbidity assessment. It is not clear why for each scale or tools the author’s used the Cronbach’s alpha test to evaluate the reliability. Please explain. | Thank you for your comment.  Since the tools were being utilized for the first time in research in Palestine, their content validity and dependability were assessed through reliability tests. Notably, each tool was either available in its Arabic version or had its translation into Arabic prior to its use. Moreover, due to the fact that each scale assesses a unique variable, reliability tests were conducted individually for each scale. | The following paragraph has been added in the methodology part (highlighted in yellow):  The study conducted reliability tests for the majority of instruments. Given that the tools were being employed for the first time in research conducted in Palestine, their content validity and dependability were evaluated using reliability testing. Significantly, every tool was either accessible in its Arabic version or had been translated into Arabic before being utilized. Furthermore, as each scale evaluates a distinct variable, reliability tests were performed separately for each scale. |
|  | 3. In the methods Authors indicate that the Charlson comorbidities index includes 17 comorbidities, why Authors include 20 comorbid conditions in the table? Please explain or correct. | Thank you for your comment.  The Charlson Comorbidities Index is containing 17 items, but three items are divided into two subitems making them 20 instead of 17. These items are diabetes and diabetes with end organ damage, mild liver disease and severe liver disease, cancer and metastatic solid tumor. | The following paragraph has been edited (highlighted in yellow):  The original Charlson index used in this study included 17 comorbidities with dichotomous responses (yes and no). Among these, three comorbid conditions were mutually exclusive: diabetes with chronic complications and diabetes without chronic complications; mild liver disease and moderate or severe liver disease; and any malignancy and metastatic solid tumor [25]. |
|  | 4. What the “assigned weighing” means? | Thank you for your comment.  The assigned weight is an integer from 1 to 6 attached to each comorbid condition, with a weight of six representing the most severe morbidity. | The following sentence has been edited under Table 2 (highlighted in yellow):  Weighting of each variable as in the Charlson Comorbidity Index from 1-6, with a weight of six representing the most severe morbidity [R1]. |
|  | 5. Table 3 includes Patient’s biochemical levels presented in n (%). The number of subjects is different for each parameter. Why have the Authors choice to include these parameters (albumin, hemoglobin, total protein, and c-reactive protein)? and why Authors report these data only in few patients? Have these parameters an association with specific cancer? Please explain the reason for your choice. Did the Authors consider that these parameters are involved in Taste alteration or malnutrition? Please explain. If there is no specific reason, or any association with the study I would suggest removing these data. | Thank you for your comment.  The number of subjects for each parameter in the biochemical data varied according to the available tests in the hospital records which differentiate from patient to other and from one cancer to another one.  In addition, (albumin, hemoglobin, total protein, and c-reactive protein) are used to assess the patients’ overall condition and nutritional status. | The following paragraph has been added (highlighted in yellow):  Biochemical data for albumin, hemoglobin, total protein, and C-reactive protein were obtained from patient recent records. These measurements were used to assess the patient's overall condition and nutritional status [R2]. |
|  | 6. The name Table 4 is used twice (line 176 and line 184) please correct it. | Thank you for your comment.  Revised and corrected. | The table name has been corrected to Table 5 (highlighted in yellow). |
|  | 7. Regarding Table 4 Chemotherapy side effects. I would suggest indicating the side effects in order of greatest percentage of frequency. | Thank you for your suggestion. Revised and corrected. | The table is rearranged in the manuscript according to your recommendation after the name is edited to Table 5. |
| **2** | **Comment** | **Reply** | **Corrections on the manuscript** |
|  | 8. The MS "Taste alteration and its relationship with nutritional status among cancer patients receiving chemotherapy, cross-sectional study." is well written and well organized with an in-depth approach and including several parameters that can modify taste in cancers. | Thank you for your comment. |  |

[R1] Austin SR, Wong Y-N, Uzzo RG, Beck JR, Egleston BL. Why Summary Comorbidity Measures Such as the Charlson Comorbidity Index and Elixhauser Score Work. Med Care. 2015 Sep;53(9): e65-72.

[R2] Kesari A, Noel JY. Nutritional Assessment. In Treasure Island (FL); 2022.
