# Supplementary material for: Taste alteration and its relationship with nutritional status among cancer patients receiving chemotherapy, cross-sectional study
Source: PLoS One. 2024 May 9;19(5):e0302990. doi: 10.1371/journal.pone.0302990 (PMC11081316; doi:10.1371/journal.pone.0302990)
Supplement: Attachment — Submitted filename: Response to Reviewers2.docx [file pone.0302990.s002.docx]

Editor-in-Chief PLOS ONE Journal

Dear Editors and reviewers,

Greetings

# We would like to submit the revised files of our manuscript title:

**“Taste alteration and its relationship with nutritional status among cancer patients receiving chemotherapy, cross-sectional study.”**

Ref. No.: PONE-D-23-31394

# We kindly thank you for your time and effort invested during the review of our manuscript. We appreciate the comments and suggestions on the submitted version of our contribution. We did all the corrections suggested by the reviewers attached to this letter in point-to-point form, all the corrections have been approved by all authors. The changes in the manuscript were mentioned in the below table also they were highlighted in yellow in the manuscript for easier tracing.

# We hope that the answer letter and the improved version of the paper will clarify open issues and we are looking forward to additional feedback on our revised proposal.

# Kind regards, The authors team.

**Corresponding author:** Dr. Manal Badrasawi

Email: [m.badrasawi@najah.edu](mailto:m.badrasawi@najah.edu)

P.O. Box: 7

Address: Nutrition and Food technology department, Faculty of Agriculture and Veterinary Medicine, An-Najah National University, Nablus, Palestine

Hand phone: +972595095811

| **Reviewer 3** | | |
| --- | --- | --- |
| **Comment** | **Reply** | **Corrections on the manuscript** |
| 1. In lines 184-185: Thanks to set the unit of the value body weight (79.43±17.34! and 77.41±17.23!). It seems that the difference is not significant before and after chemotherapy. It should be remembered that the BMI is an index of corpulence and not of malnutrition. If we consider the BMI, your patients do not present malnutrition (0.83% were underweight in line 184). Moreover 70% are obese-overweight. Why? | Thank you for your comment.  Revised and corrected.  Regarding the high percentage of overweight-obese patients in our study, this could be related to several factors other than the nutritional status, including changes in body composition related to increased fluid retention, which is a common side effect of chemotherapy. In addition, it is possible that patients had pre-existing obesity or were overweight, as indicated by the non-significant difference between their body weight before and after CT in our study. Moreover, approximately fifty percent of the study participants are breast cancer patients, who usually tend to gain weight after breast cancer diagnosis. | The edit has been highlighted in yellow, and the following paragraph has been added to discussion section (highlighted in yellow):  It is worth noting that the majority of our study participants showed a high percentage of being overweight or obese. This could be related to several factors other than nutritional status, including changes in body composition related to fluid retention, which is a common side effect of chemotherapy [R1]. In addition, it is possible that patients had pre-existing obesity or were overweight, as indicated by the non-significant difference between their body weight before and after CT in our study. Moreover, approximately fifty percent of the study participants are breast cancer patients, who usually tend to gain weight after breast cancer diagnosis due to decreased energy expenditure, hormonal imbalance, and depression, according to previous findings [R2]. |
| 2. This same observation (non-significant) is noted for the malnutrition serum parameters (table 3) such as albumin (10.9%) and total proteins (14.28%). Haemoglobin and C-reactive protein is not linked to malnutrition, but to anaemia and inflammation, respectively. Thank you for giving arguments. | Thank you for your comment.  In this study, biochemical markers (albumin, total protein, hemoglobin, and CRP) were used to assess the general nutritional status, rather than specifically focusing on malnutrition. However, we refrained from engaging into a deeper debate regarding their level in order to avoid discussing aspects that are unrelated to changes in taste. | The following paragraph has been added to dissection part (highlighted in yellow):  However, in our findings, elevated levels of C-reactive protein were attributed to a systematic inflammatory state associated with poor nutritional status in cancer patients [R3]. In addition, a low level of hemoglobin is a manifestation of anemia, where its incidence increases during chemotherapy [R4]. |
| 3. I am not opposed to the MUST test, but it is admitted that it is the Nutritional Risk Index (NRI) test or Buzby index which is the most practiced. In your study we do not know the degree of malnutrition: severe or moderate. Thank you for your comment. | Thank you for your comment.  n our study, we used MUST to assess the risk of malnutrition, as shown in the results section, nutritional status assessment part: “The malnutrition universal screening tool revealed that 58 patients (48.3%) were at low risk of malnutrition, 24 (20%) were at medium risk, and 38 (31.7%) were at high risk of malnutrition (Fig 1).” | The indicated paragraph was highlighted in yellow in the manuscript. |
| 4. If we consider that cancer patients undergoing chemotherapy present malnutrition. Is this due to anorexia (linked to cytokine storm) or to altered taste perception!! | Thank you for your comment.  Malnutrition during chemotherapy can result from a combination of factors, such as anorexia induced by cytokines, appetite loss, taste alteration induced by chemotherapy, and the chemotherapy-induced side effects. It is challenging to blame a single factor; however, according to our findings, taste alteration is a contributing factor, though not the only one associated with malnutrition during chemotherapy. Nevertheless, further studies are necessary to obtain more comprehensive results. | The following paragraph has been added to dissection part (highlighted in yellow):  Malnutrition during chemotherapy is attributed to metabolic changes such as inflammation, increased catabolism, anabolic resistance, and antineoplastic side effects like anorexia, nausea, and vomiting [R5]. However, according to our findings, taste alteration is a contributing factor in developing malnutrition, though not the only one. Nevertheless, further studies are necessary to obtain more comprehensive results. |

[R1] Rockson SG, Keeley V, Kilbreath S, Szuba A, Towers A. Cancer-associated secondary lymphoedema. Nat Rev Dis Prim. 2019;5. doi:10.1038/s41572-019-0072-5

[R2] Schvartsman G, Gutierrez-Barrera AM, Song J, Ueno NT, Peterson SK, Arun B. Association between weight gain during adjuvant chemotherapy for early-stage breast cancer and survival outcomes. Cancer Med. 2017;6: 2515–2522. doi:10.1002/cam4.1207

[R3] Yu JM, Yang M, Xu HX, Li W, Fu ZM, Lin Y, et al. Association Between Serum C-Reactive Protein Concentration and Nutritional Status of Malignant Tumor Patients. Nutr Cancer. 2019;71: 240–245. doi:10.1080/01635581.2018.1524019

[R4] Wondimneh B, Setty SAD, Asfeha GG, Belay E, Gebremeskel G, Baye G. Comparison of hematological and biochemical profile changes in pre-and post-chemotherapy treatment of cancer patients attended at ayder comprehensive specialized hospital, mekelle, northern ethiopia 2019: A retrospective cohort study. Cancer Manag Res. 2021;13: 625–632. doi:10.2147/CMAR.S274821

[R5] Baracos VE. Cancer-associated malnutrition. Eur J Clin Nutr. 2018;72: 1255–1259. doi:10.1038/s41430-018-0245-4
